# Polypharmacy Patterns in Multimorbid Older People with Cardiovascular Disease: Longitudinal Study

**DOI:** 10.3390/geriatrics7060141

**Published:** 2022-12-13

**Authors:** Noemí Villén, Albert Roso-Llorach, Carlos Gallego-Moll, Marc Danes-Castells, Sergio Fernández-Bertolin, Amelia Troncoso-Mariño, Monica Monteagudo, Ester Amado, Concepción Violán

**Affiliations:** 1 Medicines Area and Pharmacy Service, Barcelona Territorial Management, Institut Català de la Salut, 08015 Barcelona, Spain; 2 Department of Pediatrics, Obstetrics, Gynecology, and Preventive Medicine, Universitat Autònoma de Barcelona, Bellaterra, 08193 Cerdanyola del Vallès, Spain; 3 IDIAP Research Institute, 08007 Barcelona, Spain; 4 Germans Trias i Pujol Research Institute (IGTP), Camí de les Escoles, s/n, 08916 Badalona, Spain; 5 Sant Quirze del Vallès Primary Health Care Center Av. d′Ègara, s/n, Sant Quirze del Vallès, 08192 Barcelona, Spain; 6 North Metropolitan Research Support Unit, IDIAP Research Institute, Mataró, 08303 Barcelona, Spain; 7 North Metropolitan Primary Health Care Administration, Institut Català de Salut, Ctra. de Barcelona, 473, Sabadell, 08204 Barcelona, Spain

**Keywords:** polypharmacy, multimorbidity, elderly, cardiovascular disease, chronic disease, combined patterns, clustering, primary healthcare

## Abstract

(1) Introduction: Cardiovascular disease is associated with high mortality, especially in older people. This study aimed to characterize the evolution of combined multimorbidity and polypharmacy patterns in older people with different cardiovascular disease profiles. (2) Material and methods: This longitudinal study drew data from the Information System for Research in Primary Care in people aged 65 to 99 years with profiles of cardiovascular multimorbidity. Combined patterns of multimorbidity and polypharmacy were analysed using fuzzy c-means clustering techniques and hidden Markov models. The prevalence, observed/expected ratio, and exclusivity of chronic diseases and/or groups of these with the corresponding medication were described. (3) Results: The study included 114,516 people, mostly men (59.6%) with a mean age of 78.8 years and a high prevalence of polypharmacy (83.5%). The following patterns were identified: *Mental*, *behavioural*, *digestive and cerebrovascular*; *Neuropathy*, *autoimmune and musculoskeletal*; *Musculoskeletal*, *mental*, *behavioural*, *genitourinary*, *digestive and dermatological*; *Non-specific*; *Multisystemic*; *Respiratory*, *cardiovascular*, *behavioural and genitourinary*; *Diabetes and ischemic cardiopathy*; and *Cardiac*. The prevalence of overrepresented health problems and drugs remained stable over the years, although by study end, cohort survivors had more polypharmacy and multimorbidity. Most people followed the same pattern over time; the most frequent transitions were from *Non-specific* to *Mental*, *behavioural*, *digestive and cerebrovascular* and from *Musculoskeletal*, *mental*, *behavioural*, *genitourinary*, *digestive and dermatological* to *Non-specific*. (4) Conclusions: Eight combined multimorbidity and polypharmacy patterns, differentiated by sex, remained stable over follow-up. Understanding the behaviour of different diseases and drugs can help design individualised interventions in populations with clinical complexity.

## 1. Introduction

The ageing population has emerged as a major and growing challenge for public healthcare systems, with the population aged over 60 years expected to double between 2020 and 2050, and the population over 80 years of age expected to triple [1].

Cardiovascular disease (CVD) is one of the primary causes of morbidity and mortality and mainly affects people over 70 years of age [2,3]. It was responsible for 17.9 million deaths worldwide in 2019 (32% of global mortality), representing the number one cause of death in both men and women [2,3,4]. CVD is associated with multimorbidity that leads to polypharmacy (that is, actively using five or more prescription drugs), especially in people over 65 years of age, who are also more susceptible to drug-related safety problems [5,6,7]. The increased use of medication has an impact on medication-related outcomes [8] as well as on morbidity, hospitalizations, mortality, and health system expenditure [6,9,10,11]. In addition, the pharmacokinetics and pharmacodynamics of drugs are different in older compared to younger adults due to the accumulation of cellular and molecular damage, which can make organs such as the kidneys, liver, heart, or lungs more vulnerable [12,13]. Thus, polypharmacy has been identified as a priority for health systems, especially in older people [14].

At the same time, numerous studies have identified consistent patterns of cardiovascular multimorbidity, regardless of population characteristics and the analytical technique used [15,16,17,18,19,20,21,22,23]. Our group previously found that among the main patterns of multimorbidity, those dominated by CVD were associated with more advanced age along with greater mortality, polypharmacy, comorbidity, and alterations in kidney and liver function [19]. The cases that fit into these patterns were also the ones associated with the most visits to primary health care (PHC) [19,20]. Therefore, substantial health system resources are required to meet the needs of people with patterns of cardiovascular multimorbidity.

Interventions for preventing and treating the main cardiovascular risk factors have contributed to a decrease in cardiovascular mortality in Europe, although this is still considered insufficient [5,24]. However, people with CVD also have other comorbidities requiring pharmacological management. The joint prescription of drugs indicated for CVD and other comorbidities hinders optimal disease management in these people and favors side effects produced by polypharmacy [25].

The literature mainly analyses polypharmacy descriptively, in terms of drugs used in different multimorbidity patterns, and researchers focus almost exclusively on either multimorbidity or polypharmacy patterns, without simultaneously considering both. Medication is considered a proxy variable for the disease, so analysing multimorbidity and polypharmacy together can lead to overestimation errors. However, analysing multimorbidity according to the drugs indicated for treating each disease can help identify subgroups of patients who need personalized clinical management. Therefore, a joint approach to managing both disease and polypharmacy is needed; this must be personalized and appropriate for each multimorbidity and polypharmacy pattern in the subgroup of older people with CVD.

CVDs are chronic diseases, so they need to be analysed longitudinally, using continuous or repeated measurements that help characterize groups of individuals over time, according to the diseases they have, and the drugs indicated for their management. Longitudinal designs are thus especially useful for evaluating the temporal relationship that risk factors have with disease development and treatment outcomes [26], including, for example, the relationship(s) between different diseases and drugs in people with CVD.

Although there are some clinical practice guidelines with recommendations on multimorbidity and polypharmacy, they do not consider the previous medication prescribed over time and how it can influence patient management [10,25,27]. Knowing how diseases cluster in this population and the specific medications that are used continuously and together can contribute to better health care.

This study aims to identify and analyse the evolution of combined multimorbidity and polypharmacy patterns in people aged 65 years and older with CVD in Catalonia (Spain), between 2012 and 2016.

## 2. Materials and Methods

### 2.1. Design, Setting, and Population

This longitudinal study was performed in Catalonia (Spain), a Mediterranean region with an estimated population of 7,515,398 inhabitants in 2012 (study baseline) [28]. Universal health coverage is organized through the Spanish National Health Service and financed primarily by tax revenue. In Catalonia, the Catalan Health Institute is the main public healthcare provider and manages 285 PHC centres, which covered around 5,501,784 people (77.2% of Catalan population) at study baseline [29].

**Figure 1:**
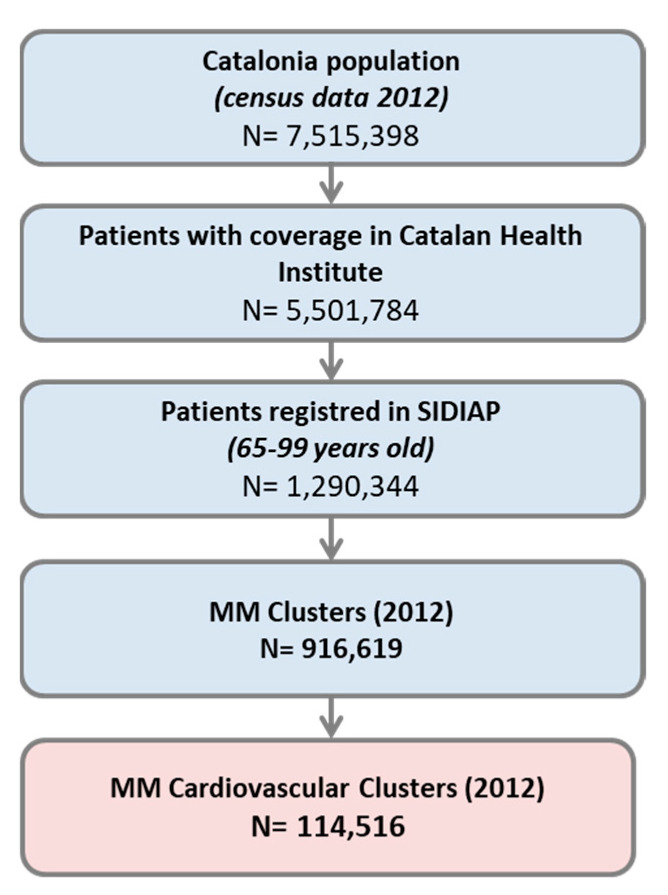
Estimated population study according to selection criteria.

Inclusion criteria were people aged 65 to 99 years in December 2011 who were alive in December 2012, had at last one visit to public PHC during the five-year study period (2012–2016), were registered in the Information System for Research in Primary Care (SIDIAP), and had been identified as belonging to two baseline cardiovascular multimorbidity patterns in a previous study [19]. No new entries were allowed in the study. Dropouts were due to mortality or transfer to another healthcare provider. A total of 114,516 individuals were included at baseline, and 72,091 completed follow-up (Figure 1).

### 2.2. Data Source

The SIDIAP database has held anonymized electronic health records (EHR), including from public PHC centres, since 2006 and was the source for all study variables. The database includes EHRs from primary and secondary health care, with data on demographics, socioeconomic status, symptoms, diagnoses, blood test results, drug prescriptions, and drug invoice information [30].

### 2.3. Variables

#### 2.3.1. Chronic Diseases and Multimorbidity

Diseases in the SIDIAP database are coded according to the International Classification of Diseases, 10th revision (ICD-10). An operational definition of multimorbidity was adopted, as described by the Swedish National study of Ageing and Care in Kungsholmen (SNAC-K) [31]. Multimorbidity was defined as the presence of two or more chronic diseases included in the 60 selected chronic disease categories.

#### 2.3.2. Drugs and Classification

Drugs registered in the SIDIAP database included medicines dispensed in community pharmacies and invoiced through the National Health Service. Drugs received in hospital and/or dispensed by a hospital pharmacy, together with all other drugs not publicly subsidized, were excluded from the study. Drugs were coded according to the Anatomical Therapeutic Chemical (ATC) Classification System [32], which categorizes drugs into increasingly specific groups according to the targeted organ/system and their chemical, pharmacological, and therapeutic properties. Altogether, 89 drug categories used in at least 1% of the study population at baseline were included. Chronic use was defined as recorded invoices for three or more packages of the same drug category during the study period. Each drug category was included as an individual dichotomous variable, and polypharmacy was likewise defined via a dichotomous variable as chronic use by the same individual of five or more different drug categories (ATC 4th level) out of the 89 drug categories.

#### 2.3.3. Drug Groups and Chronic Disease Mapping

All 89 drug categories were mapped onto the 60 SNAC-K chronic disease categories for which they are prescribed to treat. Chronic disease-drug categories were then created in the form of dichotomous variables for individuals who were diagnosed with SNAC-K chronic disease categories [33]. Fourteen categories of chronic disease require non-pharmacological treatments or treatment with drugs excluded from this study, so these could not be mapped, leaving 46 SNAC-K chronic disease categories mapped to drug categories, plus 14 chronic disease groups. Only 41 of these with a median prevalence of at least 2% over follow-up were analysed (see Supplementary Table S1).

#### 2.3.4. Analytical Variables

Over the five years of follow-up, EHRs were reviewed to identify alterations in blood test results, based on the reference values defined by Catalan Health Institute laboratories. The variables analysed were related to: kidney function—creatinine, estimated glomerular filtration rate (eGFR); liver function—aspartate aminotransferase (AST or GOT), alanine aminotransferase (ALT or GPT), gamma-glutamyltransferase (GGT), alkaline phosphatase (AP); cardiovascular risk factors—total cholesterol, blood glucose, glycosylated haemoglobin (HbA1c); proteins such as alpha-1-antitrypsin (AAT) or albumin; and variables such as haemoglobin (Supplementary Table S2).

#### 2.3.5. Demographic and Socioeconomic Variables

Demographic data, such as data on chronic drugs and diseases, were collected at baseline and monitored over follow-up. The following variables were analysed: mean age, sex (dichotomized as men or women), number of visits to PHC, and socioeconomic status. This last variable was categorized according to the Spanish MEDEA index, which classifies people into quintiles according to the level of socioeconomic deprivation in their urban area of residence (higher score = more deprivation), with a specific category for rural areas [34].

### 2.4. Statistical Analysis

The characteristics of included people were described at baseline and at each year of follow-up. Quantitative variables were described as means (standard deviation, SD) or medians (interquartile range, IQR), as appropriate, and categorical variables as frequency and percentage.

The combined multimorbidity and polypharmacy patterns were identified using a two-step approach. First, the dataset was pre-processed through a principal component analysis for categorical and continuous data (PCAmix), reducing the size and maintaining the original longitudinal data complexity [35]. To prevent statistical noise and spurious findings, only groups of diseases/drugs that achieved a median prevalence of 2% across all follow-up waves were included (Supplementary Table S1).

To select the optimal number of dimensions, the Karlis–Saporta–Spinaki rule was used [36]. For the initial clustering of participants, combined patterns based on disease/drug groups, age and sex were initially characterized through fuzzy c-means clustering [37].

Next, a hidden Markov model (HMM) was applied to characterize the temporal evolution of individuals and clusters. Sequential individual observations were assumed to follow a dynamic random process, and each cluster was associated with a hidden state. The set of HMM parameters was estimated by applying the Baum-Welch algorithm, and the best cluster was defined by means of the Viterbi algorithm [38]. The best mathematical model was selected after 100 iterations with different initializations for each number of clusters. The research team decided on the final number of clusters by making a compromise between the best log likelihood and the clinical significance.

Patterns were described in terms of prevalence, observed/expected ratios (O/E ratios) and exclusivity of chronic diseases. O/E ratios were calculated by dividing disease/drug group prevalence in the specific cluster by the disease prevalence in the total study population. Exclusivity was determined by dividing the number of patients with a disease/drug group in a specific cluster by the overall population with the same disease. Disease/drug groups were overrepresented and characterizing the cluster when the O/E-ratio was over 2. Exclusivity values were considered to be a complementary approach [19].

Patterns were compared according to the distribution of sociodemographic, clinical, and health services variables at baseline and study end. Furthermore, laboratory test values were also compared by patterns, using the chi-square test.

Analyses were carried out using R (version 4.2.1, R Foundation for Statistical Computing, Vienna, Austria). The significance level was set at α = 0.05.

## 3. Results

**Figure 2:**
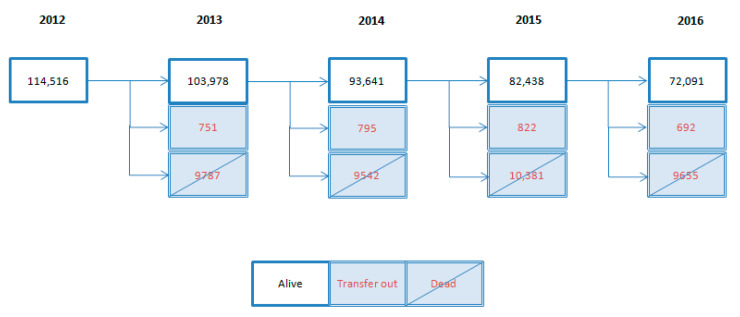
Longitudinal flow chart of study period (year 2012–2016; N = 114,516 persons).

A total of 72,091 people (63%) completed follow-up, and 39,365 people (34%) died (Figure 2).

At baseline, the people included were mostly men (59.6%), with a mean age of 78.8 years (SD 7.4) and a high prevalence (83.5%) of polypharmacy. At baseline versus study end, respectively, participants had a median of 9 (IQR 7-11) versus 10 (IQR 9-13) chronic diseases, were taking a median of 8 (IQR 6-11) medications (stable over the study period) and visited PHC annually a median of 17 (IQR 9-28) versus 18 (IQR 10-30) times (Table 1).

The chronic disease groups with the highest prevalence were those related to risk factors and CVD itself, such as hypertension, dyslipidaemia, obesity, diabetes, and heart failure. The prevalence of these diseases increased each year (Supplementary Table S1).

With respect to laboratory variables, the ones most frequently requested by physicians were total cholesterol, creatinine, and glycaemia. The percentage of people showing alterations in these indicators increased over follow-up, with glycaemia standing out as the variable with the highest number of people with one or more altered results (Table 2).

Included patients were grouped into 8 combined multimorbidity and polypharmacy patterns. The prevalence, O/E-ratio and exclusivity of the diseases, drugs and patterns for each study year are presented in Supplementary Table S3.

Patterns dominated by women included *Mental*, *behavioural*, *digestive and cerebrovascular* (62.4% women). Mental disorders, including dementia (almost exclusive to this pattern) were overrepresented (O/E-ratio > 2). Patients in this group were older and somewhat deprived. Likewise, 61.1% of the patients in the *Neuropathy*, *autoimmune and musculoskeletal* pattern were women. This pattern was characterized by a high prevalence of obesity and musculoskeletal disease, autoimmune diseases, and peripheral neuropathy. It was the pattern with the most polymedicated people and the second-youngest population. Finally, the *Musculoskeletal*, *mental*, *behavioural*, *genitourinary*, *digestive and dermatological* pattern comprised 58.6% women. This group was made up of patients with mainly rheumatic and psychiatric disorders and was characterized by a high number of diseases and medications (Table 1 and Table 3; Supplementary Table S4).

Most patients belonging to other patterns were men. In the *Non-specific* pattern (57.6% men), no disease, drug, or association was overrepresented with respect to the other patterns; it mainly includes chronic diseases that do not require pharmacological treatment, such as diseases related to the organs and senses, associated with senility and a slow disease course. This pattern was characterized by younger participants with less polypharmacy. The *Multisystemic* pattern was made up of 67.7% men. This group contained people with very varied diseases, especially digestive and chronic inflammatory diseases, and showed the highest levels of eGFR alterations. The *Respiratory*, *cardiovascular*, *behavioural and genitourinary* pattern was almost exclusively (99.2%) made up of men. It presents a high O/E-ratio and exclusivity of respiratory disease, tobacco use, and prostate disease, with the youngest population out of all the patterns. In addition, the *Diabetes and ischemic cardiopathy* pattern (66.1% men) is characterized by diabetes and ischemic heart disease. Key features include a high prevalence of obesity as well as blood glucose and Hb1A1c abnormalities (Table 2).

Finally, the *Cardiac* pattern, made up of people mostly affected by CVD, showed a similar distribution between sexes. Vascular diseases were found almost exclusively in this group, which generated the most visits to PHC out of all the patterns.

Over follow-up, the diseases and drug treatments characterizing the people grouped in each pattern remained stable, with a similar or slightly lower O/E-ratio value.

**Figure 3:**
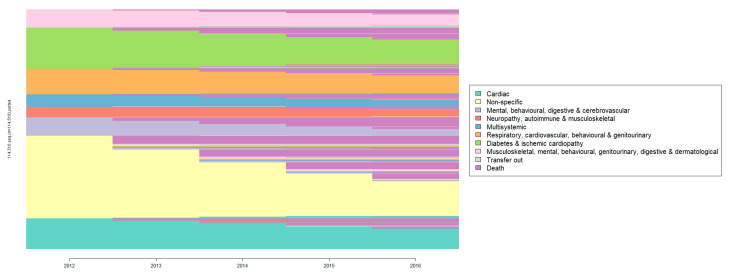
Patterns longitudinal sequences of individuals sorted by initial pattern.

Supplementary Table S4 shows the transition probability matrix between patterns. The transition matrix diagonal shows that a high proportion of patients remained in their baseline pattern throughout the follow-up period. Permanence in the same pattern was highest in the *Cardiac* pattern (67.27%) and lowest in the *Mental*, *behavioural*, *digestive and cerebrovascular* pattern (33.3%). The highest mortality was observed in *Mental*, *behavioural*, *digestive*, *and cerebrovascular* (60.2%) and *Multisystemic* (42.08%). Figure 3 shows transitions and trajectories in the individual longitudinal sequences, sorted according to the initial pattern and the number of patients in each. Most trajectories consisted of a single transition between two clusters. The most frequent transitions were to the death and dropout clusters (Supplementary Table S4). The most frequent transitions were from *Musculoskeletal*, *mental*, *behaviour*, *genitourinary*, *digestive and dermatological* to *Non-specific* (4.5%) and from *Non-specific* to *Mental*, *behavioural*, *digestive and cerebrovascular* (3.9%) (Supplementary Table S4).

## 4. Discussion

This study describes how diseases and medication are grouped in people identified in previous patterns of cardiovascular multimorbidity. Medication indicated for each disease was used as a proxy indicator for the presence of that disease, as detailed in a previous study [19]. Fuzzy c-means clustering techniques and hidden Markov models were applied in a Mediterranean population over a period of five years.

The main results of this study, which had a baseline cohort of 72,091 people aged 65 years or older, are as follows: (1) Eight multimorbidity and polypharmacy patterns were identified; (2) people with a history of CVD are subgrouped into different patterns of polypharmacy, depending on the other diseases they have; (3) multimorbidity and polypharmacy patterns are stable over time; most people remained in their baseline cluster, although some patterns were more likely to lead to death.

The multimorbidity and polypharmacy patterns identified reflect the medication most frequently administered, remaining stable over five years of follow-up. Chronic diseases such as obesity, kidney disease, hypertension, or dyslipidaemia were highly prevalent among all patterns, but they were not overrepresented in any of them. This is because all the included patients presented cardiovascular risk factors [5].

The most prevalent patterns were those mainly comprising CVDs, with a low prevalence of other diseases. These patterns were *Non-specific*, *Cardiac*, and *Diabetes and ischemic cardiopathy*.

The *Cardiac* pattern brings together two closely related diseases: atrial fibrillation and heart valve disease. Valve involvement increases the risk of thromboembolism and stroke, especially in older people and in those with associated comorbidities. The medication associated with people who have both diseases is vitamin K antagonist anticoagulants [39], which requires frequent visits to health care services to monitor medication use.

The high mortality observed in the *Mental*, *behavioural*, *digestive and cerebrovascular* pattern may be due to patients’ older age and greater multimorbidity and polypharmacy. These factors are interrelated, but also independently increase medication-related problems, worsen health outcomes, and increase mortality [6,9,10,40]. The results obtained can be interpreted in different ways. First, anaemia occurs as a complication of chronic kidney disease, caused by inadequate production of erythropoietin, associated with increased morbidity and mortality [41]. Second, chronic kidney disease has been related to an increase in dementia [42], although the exact mechanism of this relationship is unknown. Third, the overrepresented groups of diseases and medications in this cluster expose its members to various medication groups that act at the level of the central nervous system, such as antidepressants, anxiolytics, hypnotics, antipsychotics, gabapentinoids, and opioid analgesics. The concomitant use of these drugs, especially in older people, increases the risk of safety problems such as falls, respiratory depression, and death, so it is necessary to identify these people and periodically review their prescriptions to reduce their exposure to these drugs [43,44].

The *Neuropathy*, *autoimmune and musculoskeletal* pattern reflects the association between osteoporosis, osteoarthritis, and obesity. Specifically, obesity produces metabolic and inflammatory alterations that contribute to the development of osteoarthritis and osteoporosis, although these mechanisms are complex and not fully understood. Whether osteoporosis is associated with low or high body mass index is still under debate, but it is quite clear that the latter condition increases the risk of developing osteoarthritis. Bone marrow adipocytes have the same precursors as osteoblasts, which are the primary cells involved in bone formation, and the number of bone marrow adipocytes appears to be inversely related to bone mineral density. Although adipokines released by these adipocytes influence the progression of bone loss, their exact role remains controversial [45]. Pharmacological treatment for autoimmune and musculoskeletal diseases includes systemic corticosteroids, whose side effects include an increase in diabetes, especially in older and obese people [46]. This pattern, together with *Diabetes and ischemic cardiopathy*, are the ones in which more people present glycaemic alterations. Diabetes secondary to corticosteroids explains why one of the most frequent transitions is from *Neuropathy*, *autoimmune and musculoskeletal* to *Diabetes and ischemic cardiopathy*.

Diabetes is associated with more atheroma and increased macrophage infiltration and intraplaque thrombi [47]. It doubles the risk of cardiovascular disease and increases chronic kidney disease [5] helping explain the diseases overrepresented in the *Diabetes and ischemic cardiopathy* pattern.

In the *Respiratory*, *cardiovascular*, *behavioural and genitourinary* pattern, tobacco use is overrepresented (Other psychiatric and behavioural disorders). This risk factor provokes an inflammatory response and causes dysfunction in the cilia and oxidative injury in the pulmonary alveoli. It is responsible for 40% to 70% of cases of chronic obstructive pulmonary disease (COPD), which is more prevalent in men, and its association with increased prostate-specific antigen values and prostatic hyperplasia has been studied [48].

The laboratory parameters presenting the most alterations were those related to diabetes and kidney disease, two cardiovascular risk factors that figure among the 10 main causes of death [3,5,24]. Among the causes of impaired kidney function are ageing [13], diabetes [5] and the use of anti-inflammatory drugs used for musculoskeletal and inflammatory diseases. When these are administered together with drugs targeting the renin angiotensin system and diuretics used for hypertension and other CVDs, they can cause acute kidney injury (triple whammy) [49]. Consequently, we observed that the patterns in which these diseases or drugs are overrepresented (*Mental*, *behavioural*, *digestive and cerebrovascular*; *Diabetes and ischemic cardiopathy*; and *Multisystemic*) show more alterations in kidney function and higher mortality.

The trajectory of the *Non-specific* pattern presented the most changes over the study period. This result can be explained by the patient profile for this pattern, with younger individuals and fewer overrepresented diseases. As the people included in this cluster age, they acquire a greater burden of disease and more polypharmacy, moving on to more complex patterns. This results in an increase in drugs and diseases.

To the best of our knowledge, there are no similar studies to ours, as multimorbidity patterns are mainly studied independently of polypharmacy, so it is not possible to compare our results with those in the literature. Moreover, we did not find any longitudinal studies analysing polypharmacy [50]. Despite the novel approach, the same non-specific and multisystemic patterns identified by other authors, who used different clustering techniques, were replicated here [16,51,52].

This paper analysed people with high mortality who had previously been grouped into multimorbidity patterns dominated by different CVDs, unlike other studies focusing on one cardiovascular disease or type of disease, precluding comparison [53].

This study has both strengths and limitations. Among the strengths is the use of a large, high-quality database containing primary care records that are representative of the population with multimorbidity and polypharmacy in Catalonia [54]. In addition, we used a clinically oriented and validated methodology to measure chronic diseases, which allows a standardized assessment of chronic diseases in the European Union [31]. The pre-selection of people clustered in CVD patterns with a high mortality risk is a novel aspect that enables a better characterization of disease and polypharmacy subclusters. Finally, the longitudinal design was apt for evaluating the relationship between cardiovascular risk factors, the development of disease, and treatment outcomes over time [26].

Regarding the statistical analysis, different initializations can be considered in the HMM, and there is no guarantee of reaching a global optimal solution, since the HMM obtains a local optimum instead. To minimize this effect and ultimately use the model with the highest probability, we performed 100 Baum-Welch iterations with different initializations. In addition, the HMM performs better over longer periods of time. To address this limitation and increase the reliability of the model, data from all patients for each time interval were used [55].

Among the limitations, the SIDIAP database contains billing information for drugs prescribed in primary and specialized care, but not drugs used or dispensed in hospital, nor those not financed by the public healthcare system. There is also no information on people who transferred to private healthcare providers, although these would represent a minority of patients, and moreover, people receiving private healthcare services often also receive care through the public system. Furthermore, relatively few variables could be analysed, although those of the most interest for studying CVD were available. It is understood that when doctors do not request certain laboratory tests, it is because the patient′s clinical situation does not demand it, so the absence of laboratory data should not be considered a bias. The results of the study are applicable to the study period. However, recent studies carried out in Catalonia [56,57] and other countries [58] have shown that the incidence in the diagnosis of chronic diseases and cancer has decreased after the pandemic.

Knowing how diseases and medications are grouped and evolve in older people with cardiovascular pathology has implications for clinical practice. The results of the study will allow the design of more specific clinical practice guidelines and prevention strategies, leading to a more individualised management of pathologies and safer and more appropriate use of medications.

Our work exposes some topics that would be worth to investigate further in the future, suggesting that using fuzzy c-means clustering techniques and Hidden Markov models identified multimorbidity patterns and phenotypes of certain subgroups of patients that were more consistent with clinical practice. First, these results open the door to further research in subgroups of women and in younger patients. Second, our method can be applied to other prevalent diseases to find out how multimorbidity and polypharmacy patterns evolve.

## 5. Conclusions

Using HMM to obtain longitudinal patterns of multimorbidity in people with CVD allowed a characterization of this population according to their diseases and pharmacological treatments. This approach was useful for classifying patients into specific subgroups with both CVD and other prevalent diseases. Altogether, eight clinically meaningful clusters were identified based on the diseases and medications included in each. The trajectories of these multimorbidity and polypharmacy patterns were stable over time, with most people remaining in their baseline pattern, although some patterns presented a more frequent evolution towards death.

An understanding of the characteristics of these patterns and how they evolve can inform the development and implementation of strategies that contribute to providing these patients with better and more personalized health care. This study opens the door to further research in subgroups of people with high mortality. Moreover, it contributes to a deeper clinical understanding of multimorbid older people with CVD. The complexity in these patients entails a higher risk of hospital admissions, functional impairments, and mortality, and they could benefit from tailored health and social care [59]. If the associations between diseases and drugs described in this study are considered in clinical practice guidelines and in the management of older patients, its results can contribute to planning and individualising interventions in this population group.

## Figures and Tables

**Table 1:** Variables characterizing each cluster in the study at baseline and at the end of the study (N = 114,516).

Variables	Cardiac	Non-Specific	Mental, Behavioural, Digestive and Cerebrovascular	Neuropathy, Autoimmune and Musculoskeletal	Multisystemic	Respiratory, Cardiovascular, Behavioural and Genitourinary	Diabetes and Ischemic Cardiopathy	Musculoskeletal, Mental, Behavioural, Genitourinary, Digestive and Dermatological	Overall
	N_2012_ = 14,533 (12.7%)–N_2016_ = 11,145 (15.5%)	N_2012_ = 39,530 (34.5%)–N_2016_ = 17,749 (24.6%)	N_2012_ = 8937 (7.8%)–N_2016_ = 4757 (6.6%)	N_2012_ = 5002 (4.4%)–N_2016_ = 4393 (6.1%)	N_2012_ = 5929 (5.2%)–N_2016_ = 5031 (7.0%)	N_2012_ = 12,270 (10.7%)–N_2016_ = 10,024 (13.9%)	N_2012_ = 19,601 (17.1%)–N_2016_ = 12,122 (16.8%)	N_2012_ = 8714 (7.6%)–N_2016_ = 6870 (9.5%)	N_2012_ = 114,516–N_2016_ = 72,091
Sex:									
Female	7116 (49.0%)–5524 (49.6%)	16,751 (42.4%)–6917 (39.0%)	5574 (62.4%)–2925 (61.5%)	3054 (61.1%)–2548 (58.0%)	1914 (32.3%)–1554 (30.9%)	95 (0.77%)–96 (0.96%)	6644 (33.9%)–3637 (30.0%)	5108 (58.6%)–3896 (56.7%)	46,256 (40.4%)–27,097 (37.6%)
Male	7417 (51.0%)–5621 (50.4%)	22,779 (57.6%)–10,832 (61.0%)	3363 (37.6%)–1832 (38.5%)	1948 (38.9%)–1845 (42.0%)	4015 (67.7%)–3477 (69.1%)	12,175 (99.2%)–9928 (99.0%)	12,957 (66.1%)–8485 (70.0%)	3606 (41.4%)–2974 (43.3%)	68,260 (59.6%)–44,994 (62.4%)
Patients with Multimorbidity:	14,533 (100%)–11,145 (100%)	39,524 (100.0%)–17,734 (99.9%)	8937 (100%)–4757 (100%)	5002 (100%)–4393 (100%)	5929 (100%)–5031 (100%)	122,70 (100%)–10,024 (100%)	19,601 (100%)–12,122 (100%)	8714 (100%)–6870 (100%)	114,510 (100.0%)–72,076 (100.0%)
Chronic diseases number (median, IQR):	8.00 [7.00; 10.0]–10.0 [8.00; 12.0]	7.00 [6.00; 9.00]–9.00 [7.00; 10.0]	10.0 [8.00; 11.0]–11.0 [9.00; 13.0]	11.0 [9.00; 13.0]–13.0 [11.0; 15.0]	11.0 [9.00; 13.0]–13.0 [11.0; 15.0]	9.00 [7.00; 11.0]–11.0 [9.00; 13.0]	9.00 [8.00; 11.0]–11.0 [9.00; 12.0]	11.0 [9.00; 12.0]–12.0 [10.0; 14.0]	9.00 [7.00; 11.0]–10.0 [9.00; 13.0]
Patients with Polypharmacy:	12,961 (89.2%)–10,003 (89.8%)	25,588 (64.7%)–11,785 (66.4%)	8546 (95.6%)–4516 (94.9%)	4877 (97.5%)–4284 (97.5%)	5646 (95.2%)–4848 (96.4%)	10,840 (88.3%)–9043 (90.2%)	19,013 (97.0%)–11,812 (97.4%)	8117 (93.1%)–6425 (93.5%)	95,588 (83.5%)–62,716 (87.0%)
Drugs number (median, IQR):	8.00 [6.00; 10.0]–8.00 [6.00; 10.0]	6.00 [3.00; 8.00]–6.00 [4.00; 8.00]	10.0 [7.00; 12.0]–9.00 [7.00; 11.0]	11.0 [8.00; 13.0]–11.0 [9.00; 13.0]	10.0 [8.00; 13.0]–10.0 [8.00; 13.0]	8.00 [6.00; 11.0]–9.00 [6.00; 11.0]	9.00 [7.00; 12.0]–10.0 [8.00; 12.0]	9.00 [7.00; 12.0]–9.00 [7.00; 12.0]	8.00 [6.00; 11.0]–8.00 [6.00; 11.0]
Age (Mean, SD):	76.3 (6.4)–75.2 (6.0)	80.6 (7.5)–78.4 (7.2)	84.3 (6.1)–82.1 (5.8)	76.0 (6.2)–75.1 (5.9)	80.6 (6.4)–78.8 (6.1)	74.5 (6.5)–73.5 (6.0)	77.0 (6.8)–75.6 (6.36)	79.2 (6.7)–77.8 (6.4)	78.8 (7.4)–76.8 (6.8)
Age (n, %):									
[65,70)	2678 (18.4%)–2417 (21.7%)	3931 (9.94%)–2615 (14.7%)	150 (1.68%)–127 (2.67%)	935 (18.7%)–983 (22.4%)	352 (5.94%)– 429 (8.53%)	3408 (27.8%)–3165 (31.6%)	3304 (16.9%)–2571 (21.2%)	859 (9.86%)–845 (12.3%)	15,617 (13.6%)–13,152 (18.2%)
[70,80)	7123 (49.0%)–5945 (53.3%)	12,539 (31.7%)–6947 (39.1%)	1714 (19.2%)–1382 (29.1%)	2540 (50.8%)–2319 (52.8%)	2138 (36.1%)–2237 (44.5%)	6000 (48.9%)–5097 (50.8%)	9046 (46.2%)–6113 (50.4%)	3464 (39.8%)–3171 (46.2%)	44,564 (38.9%)–33,211 (46.1%)
[80,90)	4498 (31.0%)–2710 (24.3%)	18,578 (47.0%)–7212 (40.6%)	5356 (59.9%)–2830 (59.5%)	1480 (29.6%)–1073 (24.4%)	3011 (50.8%)–2195 (43.6%)	2685 (21.9%)–1713 (17.1%)	6690 (34.1%)–3289 (27.1%)	3897 (44.7%)–2671 (38.9%)	46,195 (40.3%)–23,693 (32.9%)
[90,99]	234 (1.61%)–73 (0.66%)	4482 (11.3%)–975 (5.49%)	1717 (19.2%)–418 (8.79%)	47 (0.94%)–18 (0.41%)	428 (7.22%)–170 (3.38%)	177 (1.44%)–49 (0.49%)	561 (2.86%)–149 (1.23%)	494 (5.67%)–183 (2.66%)	8140 (7.11%)–2035 (2.82%)
MEDEA *:									
R	2961 (22.6%)–2228 (20.6%)	7996 (23.0%)–3406 (19.9%)	1955 (27.3%)– 980 (21.6%)	764 (17.0%)–636 (15.0%)	1141 (22.7%)–946 (19.3%)	1866 (16.9%)–1461 (15.1%)	3368 (19.1%)–2035 (17.3%)	1507 (19.7%)–1164 (17.5%)	21,558 (21.4%)–12,856 (18.4%)
U1	1974 (15.1%)–1706 (15.8%)	5718 (16.5%)–2786 (16.3%)	1255 (17.5%)–824 (18.2%)	489 (10.9%)–490 (11.5%)	722 (14.4%)–744 (15.2%)	1558 (14.1%)–1409 (14.5%)	2487 (14.1%)–1690 (14.3%)	1188 (15.5%)–1066 (16.1%)	15,391 (15.3%)–10,715 (15.4%)
U2	2100 (16.0%)–1797 (16.6%)	5412 (15.6%)–2793 (16.3%)	1093 (15.3%)–710 (15.7%)	656 (14.6%)–633 (14.9%)	701 (13.9%)–761 (15.6%)	1714 (15.5%)–1565 (16.1%)	2638 (15.0%)–1811 (15.4%)	1176 (15.4%)–1089 (16.4%)	15,490 (15.4%)–11,159 (16.0%)
U3	1983 (15.1%)–1668 (15.4%)	5638 (16.2%)–2879 (16.8%)	1048 (14.6%)–727 (16.1%)	748 (16.7%)–738 (17.4%)	761 (15.1%)–818 (16.7%)	1797 (16.3%)–1609 (16.6%)	2953 (16.8%)–2009 (17.0%)	1225 (16.0%)–1092 (16.5%)	16,153 (16.0%)– 11,540 (16.6%)
U4	2087 (15.9%)–1772 (16.4%)	5203 (15.0%)–2774 (16.2%)	948 (13.2%)–657 (14.5%)	846 (18.8%)–797 (18.8%)	833 (16.6%)–801 (16.4%)	1978 (17.9%)–1783 (18.4%)	3050 (17.3%)–2113 (17.9%)	1248 (16.3%)–1105 (16.6%)	16,193 (16.1%)–11,802 (16.9%)
U5	1999 (15.3%)–1631 (15.1%)	4751 (13.7%)–2471 (14.4%)	866 (12.1%)–631 (13.9%)	989 (22.0%)–955 (22.5%)	871 (17.3%)–823 (16.8%)	2117 (19.2%)–1871 (19.3%)	3107 (17.7%)–2133 (18.1%)	1311 (17.1%)–1121 (16.9%)	16,011 (15.9%)–11,636 (16.7%)
**N. of visits (median, IQR):**	25.0 [15.0; 35.0]–27.0 [18.0; 36.0]	13.0 [7.00; 23.0]–13.0 [7.00; 23.0]	17.0 [10.0; 29.0]–17.0 [8.00; 29.0]	21.0 [13.0; 33.0]–22.0 [13.0; 35.0]	24.0 [14.0; 37.0]–25.0 [14.0; 39.0]	14.0 [9.00; 22.0]–15.0 [9.00; 23.0]	16.0 [10.0; 26.0]–16.0 [10.0; 26.0]	19.0 [11.0; 31.8]–20.0 [12.0; 33.0]	17.0 [9.00; 28.0]–18.0 [10.0; 30.0]

* MEDEA index starts with U1 (urban setting, least deprived) and ends with U5 (urban setting, most deprived). Individuals in rural settings are classified in the variable R.

**Table 2:** Average of analytical determinations and patients by pattern with at least one adverse result.

Year of Follow-up	Analytical Variables	Cardiac (N = 14,533)		Non-Specific (N = 39,530)		Mental, Behavioural, Digestive and Cerebrovascular (N = 8937)		Neuropathy, Autoimmune and Musculoskeletal (N = 5002)		Multisystemic (N = 5929)		Respiratory, Cardiovascular, Behavioural and Genitourinary (N = 12,270)		Diabetes and Ischemic Cardiopathy (N = 19,601)		Musculoskeletal, Mental, Behavioural, Genitourinary, Digestive and Dermatological (N = 8714)		All Population (N = 114,516)	
		Patients with Determination (%)	Patients with ≥1 Altered Determination (N, %)	Patients with Determination (%)	Patients with ≥1 Altered Determination (N, %)	Patients with Determination (%)	Patients with ≥1 Altered Determination (N, %)	Patients with Determination (%)	Patients with ≥1 Altered Determination (N, %)	Patients with Determination (%)	Patients with ≥1 Altered Determination (N, %)	Patients with Determination (%)	Patients with ≥1 Altered Determination (N, %)	Patients with Determination (%)	Patients with ≥1 Altered Determination (N, %)	Patients with Determination (%)	Patients with ≥1 Altered Determination (N, %)	Patients with Determination (%)	Patients with ≥1 Altered Determination (N, %)
2012	ALB_ser	11.3%	549 (3.78%)	10.9%	1288 (3.26%)	19.3%	340 (3.80%)	12.5%	222 (4.44%)	16.1%	295 (4.98%)	10.3%	511 (4.16%)	12.4%	856 (4.37%)	12.0%	315 (3.61%)	12.2%	4376 (3.82%)
	TCHOL	70.8%	841 (5.79%)	64.8%	2267 (5.73%)	74.2%	582 (6.51%)	81.1%	381 (7.62%)	74.6%	315 (5.31%)	73.0%	568 (4.63%)	80.4%	972 (4.96%)	73.0%	711 (8.16%)	71.7%	6637 (5.80%)
	CREAT	71.7%	1557 (10.7%)	66.0%	3848 (9.73%)	75.3%	1562 (17.5%)	81.3%	699 (14.0%)	76.9%	1268 (21.4%)	73.7%	1046 (8.52%)	80.5%	3331 (17.0%)	74.1%	833 (9.56%)	72.6%	14,144 (12.4%)
	ALP	26.2%	1034 (7.11%)	21.1%	1963 (4.97%)	24.3%	582 (6.51%)	26.7%	300 (6.00%)	29.0%	499 (8.42%)	24.1%	558 (4.55%)	24.4%	1124 (5.73%)	25.5%	508 (5.83%)	23.9%	6568 (5.74%)
	GGT	47.5%	2070 (14.2%)	43.3%	3053 (7.72%)	49.2%	699 (7.82%)	51.4%	550 (11.0%)	51.9%	887 (15.0%)	49.2%	1174 (9.57%)	51.0%	1839 (9.38%)	49.6%	782 (8.97%)	47.5%	11,054 (9.65%)
	GLYC	72.0%	6000 (41.3%)	66.5%	13,501 (34.2%)	75.9%	3718 (41.6%)	82.6%	3023 (60.4%)	76.9%	2711 (45.7%)	74.6%	5501 (44.8%)	82.0%	12,696 (64.8%)	74.3%	2953 (33.9%)	73.3%	50,103 (43.8%)
	AST (or SGOT)	27.1%	323 (2.22%)	23.6%	599 (1.52%)	26.1%	152 (1.70%)	29.1%	79 (1.58%)	30.2%	157 (2.65%)	27.0%	248 (2.02%)	27.7%	381 (1.94%)	27.2%	121 (1.39%)	26.2%	2060 (1.80%)
	ALT (or SGPT)	63.9%	631 (4.34%)	59.3%	1254 (3.17%)	67.9%	235 (2.63%)	72.3%	219 (4.38%)	68.6%	294 (4.96%)	67.4%	634 (5.17%)	70.8%	951 (4.85%)	67.6%	261 (3.00%)	65.1%	4479 (3.91%)
	HbA1c	33.5%	2745 (18.9%)	29.1%	6298 (15.9%)	42.6%	2304 (25.8%)	59.6%	2200 (44.0%)	40.9%	1422 (24.0%)	39.2%	2936 (23.9%)	64.8%	9714 (49.6%)	28.8%	1104 (12.7%)	39.8%	28,723 (25.1%)
	eGFR	58.7%	3536 (24.3%)	52.9%	8358 (21.1%)	64.0%	3530 (39.5%)	69.5%	1613 (32.2%)	64.8%	2377 (40.1%)	62.2%	1874 (15.3%)	68.1%	7042 (35.9%)	62.3%	2077 (23.8%)	60.1%	30,407 (26.6%)
2016	ALB_ser	18.6%	713 (6.40%)	15.7%	776 (4.37%)	25.9%	214 (4.50%)	23.3%	323 (7.35%)	24.2%	349 (6.94%)	19.4%	708 (7.06%)	21.3%	815 (6.72%)	19.4%	395 (5.75%)	19.7%	4293 (5.95%)
	TCHOL	75.0%	451 (4.05%)	68.7%	821 (4.63%)	73.8%	261 (5.49%)	82.7%	227 (5.17%)	77.1%	194 (3.86%)	77.0%	321 (3.20%)	81.0%	393 (3.24%)	75.9%	412 (6.00%)	75.4%	3080 (4.27%)
	CREAT	77.7%	1562 (14.0%)	71.3%	1897 (10.7%)	77.0%	830 (17.4%)	84.5%	823 (18.7%)	80.5%	1305 (25.9%)	79.7%	1245 (12.4%)	82.8%	2724 (22.5%)	78.9%	792 (11.5%)	77.9%	11,178 (15.5%)
	ALP	30.0%	861 (7.73%)	23.2%	879 (4.95%)	23.8%	316 (6.64%)	32.6%	372 (8.47%)	33.9%	537 (10.7%)	29.9%	582 (5.81%)	29.5%	845 (6.97%)	28.1%	428 (6.23%)	28.1%	4820 (6.69%)
	GGT	53.6%	1883 (16.9%)	47.1%	1524 (8.59%)	49.2%	435 (9.14%)	56.8%	613 (14.0%)	56.9%	905 (18.0%)	56.3%	1116 (11.1%)	55.0%	1298 (10.7%)	53.1%	805 (11.7%)	52.7%	8579 (11.9%)
	GLYC	77.4%	4818 (43.2%)	71.3%	6527 (36.8%)	77.0%	1990 (41.8%)	84.8%	2714 (61.8%)	80.1%	2493 (49.6%)	80.1%	4975 (49.6%)	83.5%	8089 (66.7%)	78.6%	2618 (38.1%)	78.0%	34,224 (47.5%)
	AST (or SGOT)	26.8%	294 (2.64%)	22.5%	297 (1.67%)	24.0%	85 (1.79%)	28.8%	112 (2.55%)	30.5%	153 (3.04%)	28.6%	252 (2.51%)	28.5%	273 (2.25%)	25.8%	120 (1.75%)	26.4%	1586 (2.20%)
	ALT (or SGPT)	69.1%	367 (3.29%)	63.9%	430 (2.42%)	68.4%	117 (2.46%)	74.8%	158 (3.60%)	72.3%	186 (3.70%)	72.3%	410 (4.09%)	72.9%	460 (3.79%)	71.1%	166 (2.42%)	69.6%	2294 (3.18%)
	HbA1c	36.3%	1956 (17.6%)	30.4%	2568 (14.5%)	42.1%	1156 (24.3%)	60.1%	1742 (39.7%)	42.8%	1183 (23.5%)	43.5%	2509 (25.0%)	66.7%	5698 (47.0%)	30.5%	870 (12.7%)	42.7%	17,682 (24.5%)
	eGFR	71.9%	3951 (35.5%)	65.3%	5288 (29.8%)	72.5%	2301 (48.4%)	80.3%	1975 (45.0%)	76.3%	2706 (53.8%)	74.0%	2635 (26.3%)	78.4%	5888 (48.6%)	73.3%	2346 (34.1%)	72.6%	27,090 (37.6%)

All results have *p* < 0.05. Analytical variables with ≥ 30% altered determinations are shaded in blue. Abbreviations: AAT: Alpha-1 antitrypsin, ALB_ser: Serum Albumin, TCHOL: Total Cholesterol, CREAT: Cretinine, ALP: Alkaline Phosphatase, GGT: Gamma-Glutamyl Transpeptidase, GLYC: Glycaemia, AST (or SGOT): Aspartate Aminotransferase (serum glutamic-oxaloacetic), ALT (or SGPT): Alanine Transaminase (serum glutamic-pyruvic transaminase), HbA1c: Glycosylated Haemoglobin, eGFR: estimated Glomerular Filtration Rate.

**Table 3:** Evolution of combined multimorbidity and polypharmacy patterns associated with more mortality.

Baseline (2012)					End of Follow-Up (2016)				
Pattern	Disease or Disease-Drug Group	Prev C1	OE C1	Exc C1	Pattern	Disease or Disease-Drug Group	Prev C1	OE C1	Exc C1
Mental, behavioural, digestive and cerebrovascular	Dementia	48.96	8.67	67.69	Mental, behavioural, digestive and cerebrovascular	Dementia	57.70	8.02	52.95
	Depression mood	48.20	3.54	27.64		Other digestive (D)	17.87	4.10	27.07
	Other digestive (D)	10.43	3.42	26.66		Depression mood	49.91	3.12	20.61
	Other psychiatric and behavioural	14.05	2.71	21.17		Other psychiatric and behavioural	24.24	2.73	18.03
	Neurotic, stress and somatoform	28.43	2.64	20.61		Neurotic, stress and somatoform	29.58	2.07	13.66
	Anaemia	33.21	2.54	19.85		Anaemia	30.29	2.07	13.65
	Cerebrovascular	30.18	2.04	15.92		Cerebrovascular	27.50	2.00	13.18
	Colitis related diseases	30.97	1.83	14.31		Chronic pancreas, biliary-gallbladder (D)	8.66	1.54	10.16
	Chronic pancreas, biliary-gallbladder (D)	6.99	1.83	14.25		Colitis related diseases	23.50	1.32	8.71
	Autoimmune	3.70	1.66	12.98		Chronic kidney	48.48	1.27	8.37
	Chronic kidney	39.45	1.51	11.79		Glaucoma	10.51	1.26	8.30
	Sleep	10.53	1.45	11.30		Autoimmune	3.51	1.17	7.71
	Glaucoma	10.09	1.37	10.68		Sleep	14.23	1.13	7.45
	Diabetes	39.52	1.19	9.25		Diabetes	38.28	1.09	7.21
	Osteoarthritis, degenerative joint	23.93	1.08	8.42		Thyroid	7.21	1.03	6.83
	Hypertension	80.13	1.08	8.40		Hypertension	79.40	1.01	6.68
	Osteoporosis	7.65	1.07	8.36		Osteoarthritis, degenerative joint	27.10	1.01	6.67
	Thyroid	5.96	1.06	8.25		Solid neoplasms (D)	23.10	0.97	6.39
	Solid neoplasms (D)	19.66	1.04	8.09		Osteoporosis	4.88	0.93	6.15
	Cataract lens (D)	25.09	1.02	7.93		Cataract lens (D)	29.68	0.92	6.09
Multisystemic	Chronic pancreas, biliary-gallbladder (D)	27.74	7.24	37.50	Multisystemic	Chronic pancreas, biliary-gallbladder (D)	31.01	5.51	38.45
	Inflammatory arthropathies	25.48	6.71	34.75		Inflammatory arthropathies	27.83	4.94	34.47
	Other respiratory (D)	11.01	4.76	24.63		Other respiratory (D)	13.52	3.37	23.51
	Other cardiovascular diseases (D)	17.22	3.94	20.39		Autoimmune	8.75	2.91	20.31
	Autoimmune	8.58	3.85	19.95		Other cardiovascular diseases (D)	17.21	2.89	20.17
	Other digestive (D)	9.36	3.07	15.88		Other digestive (D)	12.03	2.76	19.27
	Bradycardias conduction (D)	31.57	2.69	13.95		Bradycardias conduction (D)	38.54	2.33	16.27
	Peripheral neuropathy	10.58	2.19	11.32		Dorsopathies	23.63	1.79	12.47
	Dorsopathies	19.67	2.12	10.98		Osteoarthritis, degenerative joint	46.95	1.75	12.22
	Other genitourinary	6.53	2.03	10.48		Peripheral neuropathy	12.72	1.74	12.16
	Osteoarthritis, degenerative joint	43.11	1.94	10.06		Other genitourinary	6.98	1.73	12.05
	COPD, emphysema, chronic bronchitis	38.66	1.86	9.64		COPD, emphysema, chronic bronchitis	38.96	1.65	11.53
	Other musculoskeletal joint	15.37	1.85	9.59		Heart failure	68.79	1.63	11.35
	Chronic kidney	47.06	1.80	9.33		Anaemia	23.79	1.62	11.34
	Allergy	2.78	1.76	9.13		Other musculoskeletal joint	18.64	1.62	11.31
	Anaemia	22.43	1.72	8.89		Allergy	4.75	1.61	11.22
	Heart failure	62.35	1.71	8.83		Chronic kidney	61.30	1.60	11.20
	Other skin (D)	3.17	1.61	8.33		Sleep	18.58	1.47	10.28
	Sleep	11.62	1.60	8.28		Colitis related diseases	26.24	1.47	10.28
	Colitis related diseases	25.22	1.49	7.73		Atrial fibrillation	47.31	1.34	9.37

Categories highlighted reach the O/E-ratio threshold of two or exclusivity ≥ 30%. Abbreviations: Prev: disease prevalence in the cluster; O/E-ratio: observed/expected ratio; Exc: exclusivity; COPD: chronic obstructive pulmonary; D: Disease category (all other groups correspond to disease-drug).

## Data Availability

The datasets are not available, since researchers signed an agreement with the Information System for the Development of Research in Primary Care (SIDIAP) concerning confidentiality and security of the dataset, which forbids providing data to third parties. The SIDIAP is subject to periodic audits.

## Supplementary Materials

The following supporting information can be downloaded at: https://www.mdpi.com/article/10.3390/geriatrics7060141/s1, Table S1: Disease or disease-drug group prevalence in all population; Table S2: Values of laboratory variables determination considered altered; Table S3: Evolution of combined multimorbidity and polypharmacy patterns identified, people included and diseases or diseases-drug group; Table S4: Transition probabilities for Viterbi decoding from start to end of study between patterns.
