# Supplementary material for: Polypharmacy Patterns in Multimorbid Older People with Cardiovascular Disease: Longitudinal Study
Source: Geriatrics (Basel). 2022 Dec 13;7(6):141. doi: 10.3390/geriatrics7060141 (PMC9777651; doi:10.3390/geriatrics7060141)
Supplement: Supplementary file 1 [file geriatrics-07-00141-s001.zip › Supplementary_Table S1.pdf]

Table S1. Disease or disease-drug group prevalence in all population

Disease or Disease-Drug Group	2012		2013		2014		2015		2016		Prevalence change (%)	Prevalence median (%)
	Frequency (n)	Prevalence (%)	Frequency (n)	Prevalence (%)	Frequency (n)	Prevalence (%)	Frequency (n)	Prevalence (%)	Frequency (n)	Prevalence (%)		
Hypertension	85.263	74	78.224	75	71.530	76	63.782	77	56.540	78	5,3	76,4
Dyslipidaemia	47.958	42	44.176	42	40.642	43	36.123	44	31.639	44	4,8	43,4
Heart failure	41.860	37	40.711	39	37.979	41	34.195	41	30.480	42	15,7	40,6
Obesity (D)	39.545	35	37.550	36	34.148	36	30.811	37	27.332	38	9,8	36,5
Diabetes	38.174	33	35.185	34	32.135	34	28.521	35	25.269	35	5,1	34,3
Atrial fibrillation	33.255	29	32.188	31	30.676	33	28.126	34	25.413	35	21,4	32,8
Chronic kidney disease	29.913	26	30.390	29	29.573	32	29.651	36	27.548	38	46,3	31,6
Cataract lens (D)	28.289	25	27.539	26	26.674	28	25.054	30	23.198	32	30,3	28,5
Osteoarthritis, degenerative joint	25.400	22	23.386	22	22.742	24	21.016	25	19.335	27	20,9	24,3
Ischemic heart	28.039	24	25.413	24	22.715	24	19.920	24	17.383	24	-1,5	24,3
COPD, emphysema, chronic bronchitis	23.784	21	22.087	21	20.988	22	19.137	23	16.995	24	13,5	22,4
Prostate disease disease	22.999	20	21.722	21	20.554	22	18.904	23	17.400	24	20,2	22,0
Solid neoplasms (D)	21.721	19	21.094	20	20.123	21	18.783	23	17.208	24	25,8	21,5
Deafness hearing loss (D)	16.956	15	17.226	17	16.926	18	16.287	20	15.382	21	44,1	18,1
Colitis related diseases	19.343	17	14.033	14	13.976	15	13.472	16	12.843	18	5,4	16,3
Depression and mood diseases	15.584	14	14.939	14	14.024	15	12.680	15	11.519	16	17,4	15,0
Peripheral vascular disease	15.364	13	14.871	14	13.868	15	12.686	15	11.569	16	19,6	14,8
Cerebrovascular disease	16.940	15	15.783	15	14.008	15	12.115	15	9.925	14	-6,9	14,8
Bradycardias and conduction diseases (D)	13.423	12	13.754	13	13.543	14	12.853	16	11.916	17	41,0	14,5
Anaemia	14.954	13	13.523	13	12.843	14	11.815	14	10.556	15	12,1	13,7
Cardiac valve diseases	12.816	11	12.442	12	12.413	13	11.800	14	10.807	15	34,0	13,3
Neurotic, stress-related and somatoform diseases	12.328	11	11.920	11	11.671	12	10.969	13	10.303	14	32,7	12,5
Dorsopathies	10.615	9	10.213	10	10.364	11	10.036	12	9.537	13	42,7	11,1
Sleep disorders	8.324	7	9.268	9	9.868	11	9.700	12	9.092	13	73,5	10,5
Esophagus, stomach and duodenum diseases	9.022	8	8.902	9	9.097	10	8.879	11	8.815	12	55,2	9,7
Other musculoskeletal and joint diseases	9.499	8	9.045	9	8.992	10	8.649	10	8.292	12	38,7	9,6
Glaucoma	8.446	7	7.810	8	7.357	8	6.686	8	6.023	8	13,1	7,9
Other psychiatric and behavioral diseases	5.933	5	6.333	6	6.615	7	6.579	8	6.396	9	71,2	7,1
Dementia	6.465	6	6.378	6	6.133	7	5.619	7	5.184	7	27,3	6,6
Thyroid diseases	6.461	6	6.257	6	5.786	6	5.446	7	5.024	7	23,6	6,2
Peripheral neuropathy	5.538	5	5.422	5	5.494	6	5.477	7	5.264	7	50,8	5,9
Osteoporosis	8.178	7	6.002	6	4.991	5	4.365	5	3.772	5	-26,8	5,3
Other cardiovascular diseases (D)	5.007	4	5.038	5	4.924	5	4.684	6	4.293	6	36,2	5,3
Chronic pancreas, biliary tract and gallbladder diseases (D)	4.387	4	4.448	4	4.374	5	4.292	5	4.057	6	47,0	4,7
Inflammatory arthropathies	4.348	4	4.177	4	4.213	5	4.215	5	4.061	6	48,2	4,5
Other digestive diseases (D)	3.496	3	3.482	3	3.501	4	3.365	4	3.140	4	43,0	3,7
Other genitourinary diseases	3.691	3	3.514	3	3.179	3	3.067	4	2.913	4	25,5	3,4
Other respiratory diseases (D)	2.651	2	2.812	3	2.967	3	2.959	4	2.892	4	73,6	3,2
Autoimmune diseases	2.551	2	2.369	2	2.372	3	2.289	3	2.166	3	34,5	2,5
Other skin diseases (D)	2.258	2	2.341	2	2.366	3	2.178	3	2.018	3	42,1	2,5
Allergy	1.807	2	1.955	2	2.085	2	2.209	3	2.131	3	87,3	2,2

Abbreviations: COPD: chronic obstructive pulmonary; IQR: interquartile range. D: Disease category (all other groups correspond to disease-drug).

Categories highlighted in blue correspond to *Disease category*
