# Supplementary material for: Polypharmacy Patterns in Multimorbid Older People with Cardiovascular Disease: Longitudinal Study
Source: Geriatrics (Basel). 2022 Dec 13;7(6):141. doi: 10.3390/geriatrics7060141 (PMC9777651; doi:10.3390/geriatrics7060141)
Supplement: Supplementary file 1 [file geriatrics-07-00141-s001.zip › Supplementary_Table S2.pdf]

Table S2 . Values of laboratory variables determination considered altered

Analytical variable	Altered value	
	less than	greater than
Alpha-1 antitrypsin (AAT)	<85 mg/dL	>213 mg/dL
Serum Albumin (ALB_ser)	<3.1 g/dL	>4.3 g/dL
Total Cholesterol (TCHOL)	NA	≥6.22 mmol/L
Cretinine (CREAT)	<0.6 mg/dL	>1.5 mg/dL
Alkaline Phosphatase (ALP)	<45 IU/L	>115 IU/L
Gamma-Glutamyl Transpeptidase (GGT)	<7 IU/L	>50 IU/L
Aspartate Aminotransferase (AST or SGOT)	<4 IU/L	>40 IU/L
Alanine Transaminase (ALT or SGPT)	<0 IU/L	>40 IU/L
Glycaemia (GLYC)	<70 mg/dL	>100 mg/dL
Glycosylated Hemoglobin (HbA1c)	NA	≥6.5%
Estimated Glomerular Filtration Rate (eGFR)	<60 ml/min/1.73m^{2}	NA

NA: Does not apply
