# Supplementary material for: Polypharmacy Patterns in Multimorbid Older People with Cardiovascular Disease: Longitudinal Study
Source: Geriatrics (Basel). 2022 Dec 13;7(6):141. doi: 10.3390/geriatrics7060141 (PMC9777651; doi:10.3390/geriatrics7060141)
Supplement: Supplementary file 1 [file geriatrics-07-00141-s001.zip › Supplementary_Table S3.pdf]

Table S3. Evolution of combined multimorbidity and polypharmacy patterns identified, people included and diseases or diseases-drug group

Baseline (2012)					End of follow-up (2016)				
Pattern	Disease or Disease-Drug Group	Prev C1	OE C1	Exc C1	Pattern	Disease or Disease-Drug Group	Prev C1	OE C1	Exc C1
Cardiac	Cardiac valve diseases	64,75	5,79	73,42	Cardiac	Cardiac valve diseases	68,35	4,56	70,49
	Atrial fibrillation	88,41	3,04	38,63		Atrial fibrillation	91,77	2,60	40,25
	Heart failure	56,95	1,56	19,77		Heart failure	61,88	1,46	22,62
	Thyroid diseases	8,60	1,52	19,35		Thyroid	10,05	1,44	22,29
	Inflammatory arthropathies	5,29	1,39	17,69		Inflammatory arthropathies	7,45	1,32	20,44
	Obesity (D)	38,76	1,12	14,24		Obesity (D)	40,92	1,08	16,68
	Other cardiovascular diseases (D)	4,88	1,12	14,16		Hypertension	82,85	1,06	16,33
	Hypertension	81,17	1,09	13,83		Chronic kidney	40,07	1,05	16,21
	Osteoporosis	7,43	1,04	13,21		Osteoporosis	5,26	1,00	15,54
	Chronic kidney diseases	26,83	1,03	13,03		Chronic pancreas, biliary-gallbladder (D)	5,65	1,00	15,53
	Dyslipidaemia	40,81	0,97	12,37		Other cardiovascular diseases (D)	5,64	0,95	14,65
	Chronic pancreas, biliary-gallbladder (D)	3,65	0,95	12,10		Dyslipidaemia	41,17	0,94	14,50
	Solid neoplasms (D)	17,69	0,93	11,84		Solid neoplasms (D)	21,62	0,91	14,00
	Other skin (D)	1,71	0,87	10,98		Bradycardias conduction (D)	14,08	0,85	13,17
	Esophagus stomach duodenum	6,81	0,86	10,97		Cataract lens (D)	27,04	0,84	12,99
	COPD, emphysema, chronic bronchitis	17,06	0,82	10,43		Other skin (D)	2,30	0,82	12,69
	Bradycardias conduction (D)	9,47	0,81	10,25		COPD, emphysema, chronic bronchitis	17,93	0,76	11,76
	Anaemia	10,46	0,80	10,16		Anaemia	11,10	0,76	11,72
	Cataract lens (D)	18,33	0,74	9,42		Esophagus stomach duodenum	9,10	0,74	11,50
	Diabetes	23,57	0,71	8,97		Glaucoma	6,16	0,74	11,41
Pattern	Disease or Disease-Drug Group	Prev C1	OE C1	Exc C1	Pattern	Disease or Disease-Drug Group	Prev C1	OE C1	Exc C1
Non-specific	Bradycardias conduction (D)	14,14	1,21	41,64	Non-specific	Bradycardias conduction (D)	20,07	1,21	29,89
	Deafness hearing loss (D)	15,19	1,03	35,40		Deafness hearing loss (D)	22,18	1,04	25,59
	Other digestive (D)	3,11	1,02	35,15		Cataract lens (D)	30,01	0,93	22,96
	Cataract lens (D)	24,00	0,97	33,53		Solid neoplasms (D)	20,85	0,87	21,50
	Solid neoplasms (D)	17,00	0,90	30,95		Heart failure	35,09	0,83	20,44
	Heart failure	31,47	0,86	29,72		Other digestive (D)	3,58	0,82	20,22
	Hypertension	60,03	0,81	27,83		Hypertension	63,92	0,82	20,07
	Cerebrovascular	11,40	0,77	26,59		Osteoporosis	3,91	0,75	18,40
	Osteoporosis	5,34	0,75	25,79		Peripheral vascular	11,96	0,75	18,35
	Glaucoma	5,49	0,74	25,69		Cerebrovascular	10,24	0,74	18,32
	Peripheral vascular	9,91	0,74	25,50		Glaucoma	6,14	0,74	18,10
	Atrial fibrillation	21,34	0,73	25,37		Prostate	17,40	0,72	17,75
	Prostate	14,48	0,72	24,88		Ischemic heart	16,99	0,70	17,34
	Osteoarthritis, degenerative joint	15,79	0,71	24,57		Chronic kidney	25,94	0,68	16,71
	Obesity (D)	23,37	0,68	23,37		Atrial fibrillation	23,88	0,68	16,68
	Ischemic heart	16,56	0,68	23,35		Osteoarthritis, degenerative joint	17,60	0,66	16,16
	COPD, emphysema, chronic bronchitis	14,00	0,67	23,27		Obesity (D)	24,57	0,65	15,96
	Chronic kidney	17,18	0,66	22,71		COPD, emphysema, chronic bronchitis	14,72	0,62	15,37
	Colitis related diseases	11,08	0,66	22,64		Colitis related diseases	10,75	0,60	14,86
	Dyslipidaemia	24,92	0,60	20,54		Dyslipidaemia	26,01	0,59	14,59

Baseline (2012)					End of follow-up (2016)				
Pattern	Disease or Disease-Drug Group	Prev C1	OE C1	Exc C1	Pattern	Disease or Disease-Drug Group	Prev C1	OE C1	Exc C1
Mental, behavioural, digestive & cerebrovascular	Dementia	48,96	8,67	67,69	Mental, behavioural, digestive & cerebrovascular	Dementia	57,70	8,02	52,95
	Depression mood	48,20	3,54	27,64		Other digestive (D)	17,87	4,10	27,07
	Other digestive (D)	10,43	3,42	26,66		Depression mood	49,91	3,12	20,61
	Other psychiatric and behavioural	14,05	2,71	21,17		Other psychiatric and behavioural	24,24	2,73	18,03
	Neurotic, stress and somatoform	28,43	2,64	20,61		Neurotic, stress and somatoform	29,58	2,07	13,66
	Anaemia	33,21	2,54	19,85		Anaemia	30,29	2,07	13,65
	Cerebrovascular	30,18	2,04	15,92		Cerebrovascular	27,50	2,00	13,18
	Colitis related diseases	30,97	1,83	14,31		Chronic pancreas, biliary-gallbladder (D)	8,66	1,54	10,16
	Chronic pancreas, biliary-gallbladder (D)	6,99	1,83	14,25		Colitis related diseases	23,50	1,32	8,71
	Autoimmune	3,70	1,66	12,98		Chronic kidney	48,48	1,27	8,37
	Chronic kidney	39,45	1,51	11,79		Glaucoma	10,51	1,26	8,30
	Sleep	10,53	1,45	11,30		Autoimmune	3,51	1,17	7,71
	Glaucoma	10,09	1,37	10,68		Sleep	14,23	1,13	7,45
	Diabetes	39,52	1,19	9,25		Diabetes	38,28	1,09	7,21
	Osteoarthritis, degenerative joint	23,93	1,08	8,42		Thyroid	7,21	1,03	6,83
	Hypertension	80,13	1,08	8,40		Hypertension	79,40	1,01	6,68
	Osteoporosis	7,65	1,07	8,36		Osteoarthritis, degenerative joint	27,10	1,01	6,67
	Thyroid	5,96	1,06	8,25		Solid neoplasms (D)	23,10	0,97	6,39
	Solid neoplasms (D)	19,66	1,04	8,09		Osteoporosis	4,88	0,93	6,15
	Cataract lens (D)	25,09	1,02	7,93		Cataract lens (D)	29,68	0,92	6,09
Pattern	Disease or Disease-Drug Group	Prev C1	OE C1	Exc C1	Pattern	Disease or Disease-Drug Group	Prev C1	OE C1	Exc C1
Neuropathy, autoimmune & musculoskeletal	Peripheral neuropathy	42,48	8,78	38,37	Neuropathy, autoimmune & musculoskeletal	Peripheral neuropathy	46,28	6,34	38,62
	Autoimmune	16,39	7,36	32,14		Autoimmune	17,76	5,91	36,01
	Other musculoskeletal joint	37,45	4,51	19,72		Other musculoskeletal joint	40,97	3,56	21,71
	Dorsopathies	41,28	4,45	19,45		Dorsopathies	47,05	3,56	21,67
	Osteoarthritis, degenerative joint	62,00	2,80	12,21		Osteoarthritis, degenerative joint	66,15	2,47	15,03
	Osteoporosis	17,51	2,45	10,71		Osteoporosis	12,22	2,34	14,24
	Inflammatory arthropathies	8,20	2,16	9,43		Inflammatory arthropathies	9,88	1,75	10,69
	Obesity (D)	63,01	1,82	7,97		Diabetes	58,82	1,68	10,23
	Diabetes	59,12	1,77	7,75		Obesity (D)	63,37	1,67	10,19
	Anaemia	21,51	1,65	7,20		Anaemia	23,90	1,63	9,95
	Dyslipidaemia	60,56	1,45	6,32		Thyroid	9,74	1,40	8,52
	Depression mood	19,23	1,41	6,17		Peripheral vascular	21,83	1,36	8,29
	Peripheral vascular	18,57	1,38	6,05		Depression mood	21,56	1,35	8,22
	Neurotic, stress and somatoform	14,65	1,36	5,95		Dyslipidaemia	59,16	1,35	8,21
	Thyroid	7,60	1,35	5,88		Chronic kidney	49,51	1,30	7,90
	Chronic kidney	34,53	1,32	5,77		Neurotic, stress and somatoform	18,05	1,26	7,70
	Ischemic heart	29,71	1,21	5,30		Heart failure	51,83	1,23	7,47
	Hypertension	89,66	1,20	5,26		Ischemic heart	29,11	1,21	7,36
	Glaucoma	8,80	1,19	5,21		Glaucoma	9,74	1,17	7,11
	Heart failure	42,08	1,15	5,03		Hypertension	90,76	1,16	7,05

Baseline (2012)					End of follow-up (2016)				
Pattern	Disease or Disease-Drug Group	Prev C1	OE C1	Exc C1	Pattern	Disease or Disease-Drug Group	Prev C1	OE C1	Exc C1
Multisystemic	Chronic pancreas, biliary-gallbladder (D)	27,74	7,24	37,50	Multisystemic	Chronic pancreas, biliary-gallbladder (D)	31,01	5,51	38,45
	Inflammatory arthropathies	25,48	6,71	34,75		Inflammatory arthropathies	27,83	4,94	34,47
	Other respiratory (D)	11,01	4,76	24,63		Other respiratory (D)	13,52	3,37	23,51
	Other cardiovascular diseases (D)	17,22	3,94	20,39		Autoimmune	8,75	2,91	20,31
	Autoimmune	8,58	3,85	19,95		Other cardiovascular diseases (D)	17,21	2,89	20,17
	Other digestive (D)	9,36	3,07	15,88		Other digestive (D)	12,03	2,76	19,27
	Bradycardias conduction (D)	31,57	2,69	13,95		Bradycardias conduction (D)	38,54	2,33	16,27
	Peripheral neuropathy	10,58	2,19	11,32		Dorsopathies	23,63	1,79	12,47
	Dorsopathies	19,67	2,12	10,98		Osteoarthritis, degenerative joint	46,95	1,75	12,22
	Other genitourinary	6,53	2,03	10,48		Peripheral neuropathy	12,72	1,74	12,16
	Osteoarthritis, degenerative joint	43,11	1,94	10,06		Other genitourinary	6,98	1,73	12,05
	COPD, emphysema, chronic bronchitis	38,66	1,86	9,64		COPD, emphysema, chronic bronchitis	38,96	1,65	11,53
	Other musculoskeletal joint	15,37	1,85	9,59		Heart failure	68,79	1,63	11,35
	Chronic kidney	47,06	1,80	9,33		Anaemia	23,79	1,62	11,34
	Allergy	2,78	1,76	9,13		Other musculoskeletal joint	18,64	1,62	11,31
	Anaemia	22,43	1,72	8,89		Allergy	4,75	1,61	11,22
	Heart failure	62,35	1,71	8,83		Chronic kidney	61,30	1,60	11,20
	Other skin (D)	3,17	1,61	8,33		Sleep	18,58	1,47	10,28
	Sleep	11,62	1,60	8,28		Colitis related diseases	26,24	1,47	10,28
	Colitis related diseases	25,22	1,49	7,73		Atrial fibrillation	47,31	1,34	9,37
Pattern	Disease or Disease-Drug Group	Prev C1	OE C1	Exc C1	Pattern	Disease or Disease-Drug Group	Prev C1	OE C1	Exc C1
Respiratory, cardiovascular, behavioural & genitourinary	Allergy	8,35	5,29	56,72	Respiratory, cardiovascular, behavioural & genitourinary	Allergy	11,61	3,93	54,62
	Other respiratory (D)	9,45	4,08	43,72		Other cardiovascular diseases (D)	19,41	3,26	45,33
	Other cardiovascular diseases (D)	17,46	3,99	42,78		Other respiratory (D)	12,24	3,05	42,43
	Other psychiatric and behavioural	20,38	3,93	42,15		Other psychiatric and behavioural	23,78	2,68	37,27
	Prostate	48,33	2,41	25,78		Prostate	51,75	2,14	29,81
	COPD, emphysema, chronic bronchitis	44,63	2,15	23,02		Other genitourinary	8,14	2,01	28,01
	Peripheral vascular	27,64	2,06	22,07		COPD, emphysema, chronic bronchitis	46,31	1,96	27,31
	Other skin (D)	3,93	1,99	21,35		Peripheral vascular	31,12	1,94	26,96
	Esophagus stomach duodenum	14,58	1,85	19,83		Other skin (D)	4,97	1,77	24,68
	Sleep	12,80	1,76	18,87		Esophagus stomach duodenum	19,16	1,57	21,79
	Other genitourinary	5,22	1,62	17,34		Sleep	18,64	1,48	20,55
	Solid neoplasms (D)	29,61	1,56	16,73		Solid neoplasms (D)	34,62	1,45	20,17
	Deafness hearing loss (D)	21,30	1,44	15,42		Ischemic heart	34,50	1,43	19,89
	Neurotic, stress and somatoform	15,24	1,42	15,17		Deafness hearing loss (D)	28,93	1,36	18,85
	Ischemic heart	34,10	1,39	14,92		Colitis related diseases	24,13	1,35	18,84
	Colitis related diseases	21,77	1,29	13,81		Cerebrovascular	18,06	1,31	18,24
	Other musculoskeletal joint	10,50	1,27	13,56		Neurotic, stress and somatoform	18,51	1,29	18,00
	Cerebrovascular	18,66	1,26	13,51		Chronic pancreas, biliary-gallbladder (D)	7,15	1,27	17,67
	Inflammatory arthropathies	4,73	1,24	13,34		Dyslipidaemia	53,11	1,21	16,83
	Dyslipidaemia	51,59	1,23	13,20		Depression mood	19,00	1,19	16,54

Baseline (2012)					End of follow-up (2016)				
Pattern	Disease or Disease-Drug Group	Prev C1	OE C1	Exc C1	Pattern	Disease or Disease-Drug Group	Prev C1	OE C1	Exc C1
Diabetes & ischemic cardiopathy	Diabetes	71,00	2,13	36,45	Diabetes & ischemic cardiopathy	Ischemic heart	49,55	2,05	34,55
	Ischemic heart	50,95	2,08	35,61		Diabetes	71,14	2,03	34,13
	Peripheral vascular	26,79	2,00	34,18		Peripheral vascular	31,89	1,99	33,42
	Other genitourinary	6,41	1,99	34,06		Other genitourinary	7,25	1,79	30,18
	Thyroid	9,99	1,77	30,30		Cerebrovascular	22,90	1,66	27,97
	Glaucoma	12,73	1,73	29,54		Glaucoma	13,67	1,64	27,51
	Dyslipidaemia	72,11	1,72	29,47		Dyslipidaemia	70,79	1,61	27,12
	Cerebrovascular	23,78	1,61	27,52		Thyroid	10,68	1,53	25,78
	Other skin (D)	3,08	1,56	26,75		Other skin (D)	3,98	1,42	23,89
	Chronic kidney	39,36	1,51	25,79		Anaemia	20,31	1,39	23,32
	Anaemia	18,51	1,42	24,26		Chronic kidney	52,53	1,37	23,12
	Obesity (D)	48,11	1,39	23,85		Obesity (D)	48,90	1,29	21,69
	Hypertension	93,43	1,25	21,48		Hypertension	93,94	1,20	20,14
	Esophagus stomach duodenum	9,44	1,20	20,51		Esophagus stomach duodenum	14,20	1,16	19,52
	Prostate	21,16	1,05	18,03		Cataract lens (D)	33,81	1,05	17,67
	Cataract lens (D)	24,91	1,01	17,26		Prostate	25,32	1,05	17,64
	Solid neoplasms (D)	18,52	0,98	16,72		Solid neoplasms (D)	23,85	1,00	16,80
	Heart failure	35,58	0,97	16,66		Colitis related diseases	17,07	0,96	16,11
	Colitis related diseases	16,12	0,95	16,33		Heart failure	39,80	0,94	15,83
	COPD, emphysema, chronic bronchitis	17,86	0,86	14,72		Deafness hearing loss (D)	18,31	0,86	14,43
Pattern	Disease or Disease-Drug Group	Prev C1	OE C1	Exc C1	Pattern	Disease or Disease-Drug Group	Prev C1	OE C1	Exc C1
Musculoskeletal, mental, behavioural, genitourinary, digestive & dermatological	Neurotic, stress and somatoform	43,42	4,03	30,69	Musculoskeletal, mental, behavioural, genitourinary, digestive & dermatological	Neurotic, stress and somatoform	47,99	3,36	32,00
	Dorsopathies	34,48	3,72	28,31		Dorsopathies	38,30	2,89	27,59
	Other musculoskeletal joint	27,86	3,36	25,56		Depression mood	46,11	2,89	27,50
	Depression mood	44,53	3,27	24,90		Osteoporosis	14,57	2,78	26,54
	Sleep	23,24	3,20	24,33		Other musculoskeletal joint	31,06	2,70	25,74
	Osteoporosis	22,58	3,16	24,06		Sleep	32,34	2,56	24,44
	Other genitourinary	9,23	2,86	21,78		Osteoarthritis, degenerative joint	59,27	2,21	21,06
	Esophagus stomach duodenum	20,94	2,66	20,23		Esophagus stomach duodenum	25,62	2,10	19,97
	Osteoarthritis, degenerative joint	56,68	2,56	19,44		Other skin (D)	5,69	2,03	19,38
	Other skin (D)	4,92	2,50	19,00		Thyroid	14,08	2,02	19,25
	Thyroid	12,62	2,24	17,03		Other psychiatric and behavioural	16,49	1,86	17,71
	Allergy	3,32	2,10	15,99		Colitis related diseases	32,78	1,84	17,53
	Colitis related diseases	33,45	1,98	15,07		Other genitourinary	7,32	1,81	17,27
	Other psychiatric and behavioural	9,73	1,88	14,29		Allergy	5,01	1,69	16,14
	Peripheral neuropathy	8,77	1,81	13,80		Deafness hearing loss (D)	31,78	1,49	14,19
	Deafness hearing loss (D)	23,41	1,58	12,03		Peripheral neuropathy	10,54	1,44	13,75
	Other respiratory (D)	3,44	1,49	11,32		Other respiratory (D)	5,04	1,26	11,96
	Cataract lens (D)	33,60	1,36	10,35		Cataract lens (D)	40,00	1,24	11,85
	Heart failure	43,08	1,18	8,97		Heart failure	50,35	1,19	11,35
	COPD, emphysema, chronic bronchitis	23,74	1,14	8,70		Atrial fibrillation	39,69	1,13	10,73

Categories highlighted in red reach the O/E ratio threshold of two or exclusivity ≥30%. Abbreviations: Prev: disease prevalence in the cluster; O/E ratio: observed/expected ratio;
Exc: exclusivity; COPD: chronic obstructive pulmonary; D: Disease category (all other groups correspond to disease-drug).
Categories highlighted in blue correspond to Disease category
