# Supplementary material for: Polypharmacy Patterns in Multimorbid Older People with Cardiovascular Disease: Longitudinal Study
Source: Geriatrics (Basel). 2022 Dec 13;7(6):141. doi: 10.3390/geriatrics7060141 (PMC9777651; doi:10.3390/geriatrics7060141)
Supplement: Supplementary file 1 [file geriatrics-07-00141-s001.zip › Supplementary_Table S4.pdf]

Table S4. Transition probabilities for Viterbi decoding from start to end of study

2012 \ 2016	Cardiac	Non-specific	Mental, Behavioural, digestive & cerebrovascular	Neuropathy, autoimmune & musculoskeletal	Multisystemic	Respiratory, cardiovascular, Behavioural & genitourinary	Diabetes & ischemic cardiopathy	Musculoskeletal, mental, Behavioural, genitourinary, digestive & dermatological	Transfer out	Death
Cardiac	67,27	0,77	0,4	1,21	1,54	0,02	0,16	0,98	1,18	26,48
Non-specific	**2,59**	42,06	3,94	0,63	2,27	2,37	1,17	3,17	4,97	36,82
Mental, Behavioural, digestive & cerebrovascular	0,11	3,05	33,3	0,03	0,17	0,06	0,1	0,14	2,84	60,2
Neuropathy, autoimmune & musculoskeletal	1,8	1,09	0,32	68,11	1,4	0,13	3,86	0,3	1,1	21,9
Multisystemic	0,35	1,04	0,2	0,12	54,04	0,06	0,27	0,26	1,58	42,08
Respiratory, cardiovascular, Behavioural & genitourinary	0,11	1,13	0,13	0,07	1,1	70,39	0,94	0,35	1,08	24,69
Diabetes & ischemic cardiopathy	0,83	1,2	1,08	2,84	2,3	2,22	57,95	0,67	0,98	29,93
Musculoskeletal, mental, Behavioural, genitourinary, digestive & dermatological	1,42	4,5	1,09	0,25	1,29	0,25	0,34	60,68	1,83	28,36

Note: Patterns for 2012 are shown vertically and 2016 horizontally. Patterns shaded in grey correspont to transfer out and dead.Patterns shaded in red corrrespond to corresponds to the most frequent transitions
